# The Potential of Natural Products in Metabolic Disease Management: A Thorough Exploration of the Case of Uganda

**DOI:** 10.2174/0118715303320109241014064209

**Published:** 2025-01-10

**Authors:** Allan Amooti Ahikiriza, Sarad Pawar Naik Bukke, Tadele Mekuriya Yadesa, Buyinza Nicholas, Kasolo Daniel, Kabali Moses, Sewalu Mathias Bonny, Pranav Kumar Prabhakar

**Affiliations:** 1 Department of Pharmaceutics and Pharmaceutical Technology, Kampala International University, Western Campus, P.O. Box 71, Ishaka, Bushenyi, Uganda;; 2 Department of Clinical Pharmacy and Pharmacy Practice, Kampala International University, Western Campus, P.O. Box 71, Ishaka, Bushenyi, Uganda;; 3 Research and Development Cell, and Parul Institute of Applied Sciences, Parul University, P.O. Limda, Ta.Waghodia, Dist. Vadodara, Gujarat-391760, India

**Keywords:** Metabolic disease, natural product, ugandan healers, modern science, *Moringa oleifera*, health care routine

## Abstract

As Ugandans grapple with an increase in metabolic diseases, researchers are turning to their rich tradition of natural remedies. This review explores promising plants, such as *Moringa oleifera*, bridging the gap between the wisdom of Ugandan healers and modern science. Although these plants show potential, challenges remain. Many lack rigorous testing, standardized extracts, and long-term safety data. To unlock their true potential, a multipronged approach is needed. First, well-designed clinical trials are crucial to bringing together traditional healers and modern researchers. Imagine a Ugandan pharmacist precisely measuring a *Moringa oleifera* extract – this standardization ensures consistent results for future patients. Second, researchers need to delve deeper into how these plants influence the body. Finally, long-term safety studies are essential, especially when combined with medications. By following these steps, researchers can unleash the true power of Ugandan natural products. This empowers Ugandans to take control of their health. Future exploration of lesser-known plants and culturally sensitive education programs can further equip Ugandans on their way to well-being.

## 1 INTRODUCTION

Type-2 Diabetes Mellitus (DM) is a complex metabolic disorder characterized by progressive loss of pancreatic β- cell function and insulin receptor sensitivity [1, 2]. In low and middle-income countries (LMICs), type-2 DM is rapidly becoming an epidemic [3]. Many Ugandans are struggling with an increase in metabolic diseases, such as type 2 diabetes, obesity, and metabolic syndrome, placing a strain on the healthcare system [4-6]. Unfortunately, access to conventional medications can be limited due to cost and availability [7]. As a result, researchers are exploring complementary therapies, and natural products are emerging as a promising option [8, 9].

### 1.1 Advantages Of Natural Products

Natural products can offer several advantages over conventional medications:

• **Cost-effectiveness:** These plants are often readily available and affordable, especially valuable in resource-limited settings [10, 11].

• **Fewer side effects:** Natural products are generally perceived to have fewer side effects than conventional medications [10, 12, 13]. However, caution is advised, as some natural products can interact with medications or have their own side effects [14-16].

## 2 PROMISING NATURAL PRODUCTS IN UGANDA

Uganda has a rich tradition of using plants for medicinal purposes. Here are some examples of plants that show promise for metabolic health:

• ***Moringa oleifera*:** Moringa leaf extracts show promise in improving blood sugar control in diabetic patients [17]. It may work by lowering blood sugar and improving insulin secretion [18, 19].

• ***Garcinia kola*:** Extracts from Garcinia kola seeds have been traditionally used for weight management, and research suggests potential benefits [20, 21]. *Garcinia kola* can help by potentially inhibiting fat production and increasing satiety [22].

• ***Vernonia amygdalina* (bitter leaf):** Traditionally used for diabetes, studies suggest it may improve blood sugar control [23].

• ***Allium sativum* (garlic):** Known for its heart-healthy properties, garlic may also help regulate blood sugar [24].

• ***Zingiber officinale* (ginger):** Ginger has anti-inflammatory properties and may improve insulin sensitivity [25, 26].

Another promising plant is *Artemisia annua*, which is among the six *Artemisia spps* with the highest percentage of terpenoids, with sesquiterpene lactones (SLns) being the most predominant [27-29]. Sesquiterpene lactones (SLns) are a lipophilic sub-group of terpenoids characterized by a structural isoprenoid ring system, a lactone ring [30]. SLns are known to improve insulin sensitivity through activation of the insulin receptor substrate (IRS) and phosphorylation of Akt in skeletal muscle [26].

### 2.1 Nanoparticle Delivery Systems

Nanoparticle delivery systems are being explored as a way to improve the efficacy and bioavailability of natural products [31-33]. These systems can potentially enhance targeted delivery, improve absorption, and increase the potency of natural compounds [34-37].

Several natural products show promise for the management of metabolic diseases, with some potential mechanisms outlined below.

• **Curcumin (*Curcuma longa*):** This compound found in turmeric has anti-inflammatory and antioxidant properties. Studies suggest that curcumin can improve insulin sensitivity and regulate blood sugar levels, possibly by modulating inflammatory signaling pathways [38, 39]. However, more research is needed to determine optimal dosages and ensure long-term safety, as high doses can cause digestive side effects [38, 40, 41].

• **Berberine:** This plant alkaloid extracted from various plants holds promise in regulating blood sugar and lipid profiles [42]. Studies suggest potential benefits in the management of diabetes and cardiovascular disease, possibly by activating an enzyme adenosine monophosphate-activated protein kinase (AMPK) that improves cell glucose uptake and reduces cholesterol production in the liver [43, 44]. However, more research is needed on long-term effects and drug interactions, as berberine can interact with certain medications [4, 44].

• **Bitter Melon (*Momordica charantia*):** Recent research suggests that bitter melon may improve glycemic control, potentially offering a natural approach to diabetes management [45]. However, further research into its efficacy and safety for long-term use is warranted [36].

### 2.2 Other Promising Natural Products

Several other natural products are being explored for their potential benefits:

• **Fenugreek (*Trigonella foenum-graecum*):** Studies suggest that fenugreek seeds may improve blood sugar control and insulin sensitivity [46].

• **Green Tea (*Camellia sinensis*):** Regular green tea consumption may be associated with weight loss and improved blood sugar control [46].

• **Probiotics:** These live microorganisms show promise in modulating gut microbiota, which may improve metabolic health [47].

### 2.3 Bridging The Gap: Traditional Knowledge And Modern Medicine

Ugandan traditional medicine has a long history of using plants to treat metabolic disorders. This knowledge base is a valuable resource for scientific exploration [13, 48]. Modern research validates some of these practices, paving the way for their integration into evidence-based healthcare [49, 50]. Collaboration between traditional healers and modern healthcare providers promotes a more comprehensive approach, using both wisdom and science [51, 52].

### 2.4 Safety Considerations And Consulting A Healthcare Professional

It is vital to consult with a qualified healthcare professional before using natural products. Some may interact with medications or have their own side effects [15, 53]. Proper identification and standardization of the plant materials used are crucial to avoid contamination and ensure consistent quality and dosage [16, 50].

### 2.5 The Need For Further Research

Although the potential of natural products is promising, further research is needed.

• **Rigorous Clinical Trials:** Well-designed, randomized controlled clinical trials with standardized dosages are needed to assess efficacy and safety for specific conditions [54, 55]. These trials should determine the most effective formulations, dosages, and duration of treatment [4, 56].

• **Mechanisms of Action:** Understanding how natural products work is crucial to optimizing treatment strategies [57-59]. More research is needed to explore the detailed mechanisms by which these products influence metabolic pathways [4, 60].

• **Long-term Safety:** Long-term safety profiles, especially when used in combination with conventional medications, are needed to inform optimal dosing regimens and minimize risks [4, 10].

### 2.6 Standardized Doses for Clinical Research and Applications

Researchers need to emphasize the importance of standardized dosages in research investigating natural products, such as berberine [61, 62]. The effectiveness of these products can vary significantly depending on the concentration of their active ingredients. Standardized extracts ensure consistent delivery of these active components, allowing researchers to accurately assess their true therapeutic potential [4, 63]. This standardization will also be critical to developing safe and efficacious future clinical applications of these natural products [64, 65].

## 3 IMPORTANT CONSIDERATIONS REGARDING NATURAL PRODUCTS

Although research on natural products, such as berberine, is promising, there are limitations to consider.

### 3.1 Limitations Of Current Research

• **Limited Research:** Many natural products lack extensive clinical research to confirm their efficacy and safety for specific metabolic conditions.

• **Limited Sample Sizes:** Many studies have involved a relatively small number of participants, making it difficult to draw definitive conclusions about the efficacy.

• **Availability of Standardized Extracts:** Standardized extracts of natural products may not be readily available in all regions, especially in resource-limited settings.

• **Short Duration:** Research on many natural products has been short-term, raising questions about their long-term safety and effectiveness.

• **Diversity of Participants:** Studies often focus on specific populations, limiting the generalizability of the findings to larger populations.

### 3.2 Additional Considerations For Natural Products

• **Palatability:** Some natural products may have unpleasant tastes or require large doses in unrefined forms, preventing patient compliance.

• **Sustainability of Harvesting Practices:** The large-scale harvesting of certain plants for the production of natural products could raise sustainability concerns [66].

• **Dosage and Standardization:** The dose and standardization of natural products can vary significantly, affecting their effectiveness and safety [67, 68]. Consulting a healthcare professional for guidance on appropriate use is essential.

• **Potential Interactions:** As with any medication or supplement, natural products may interact with other medications. It is crucial to disclose all medications and supplements to your healthcare provider before incorporating natural products into your regimen [69, 70].

These limitations highlight the need for further research with larger, more diverse populations, along with the development of strategies to improve palatability, ensure accessibility of standardized extracts, and promote sustainable harvesting practices [4, 71]. By addressing these limitations and highlighting the importance of standardized dosages, research on natural products, such as berberine can move forward and contribute to improved management of metabolic diseases in Uganda and around the world [54, 72].

## CONCLUSION

Uganda is fighting a tough fight against rising metabolic diseases. However, there is a glimmer of hope brewing in their own backyard: Natural remedies. These ancient traditions, passed down through generations, offer a potential path toward more affordable and complementary treatments alongside conventional medicine.

The key lies in unlocking the true potential of these natural options through rigorous research that determines their efficacy and safety. Ugandan scientists should study the potential of the available natural products systematically and collaboratively from *in-vitro* to animal studies and ultimately in clinical uses. Scientists and Ugandan healers must unite. Together, they can bridge the gap between ancient wisdom and modern science. By conducting well-designed clinical trials and unravelling how these plants work on a cellular level, researchers can pave the way for targeted therapies and personalized treatment plans. This integrative approach has the power to not only improve population health, but also empower Ugandans to take charge of their well-being and build a healthier future, one plant, one person at a time.

## RECOMMENDATIONS

To fully harness the potential of natural products, Uganda should:

• **Invest in high-quality research:** Conduct well-designed, randomized, controlled clinical trials with standardized dosages of natural products, focusing on specific metabolic diseases prevalent in Uganda, to evaluate both efficacy and safety.

• **Unlock the Mechanisms:** Delve deeper into the mechanisms by which these natural products exert their beneficial effects on metabolic health markers. This knowledge will inform the development of targeted therapies and optimize treatment strategies.

• **Pharmacokinetic studies:** Conduct pharmacokinetic studies to understand the absorption, distribution, metabolism, and excretion of natural products within the body. This knowledge will contribute to establishing optimal dosing regimens and minimizing potential side effects.

• **Develop evidence-based guidelines:** Establish clear and comprehensive guidelines for the safe and effective integration of natural products into conventional treatment plans. These guidelines should consider potential interactions with medications and address cultural considerations for patient education and adherence.

• **Standardization is Key:** Develop standardized protocols for preparing, administering, and storing natural products to ensure consistent quality and 'dosage' and minimize contamination risks [54].

• **Long-term safety:** Further research is needed to establish the long-term safety profiles of specific natural products, particularly when used in combination with conventional medications.

## ADDITIONAL CONSIDERATIONS

**1. Invest in Education:** Implement culturally sensitive educational programs for both healthcare professionals and the public [73]. These programs should promote the safe and effective use of natural products for managing metabolic diseases [73].

Empowering Ugandans through education: 

Culturally sensitive education programs are essential for both healthcare professionals and the public; imagine a Ugandan pharmacist confidently explaining the benefits and potential side effects of a standardized *Moringa oleifera* extract to a patient [74]. Educating the public empowers them to make informed decisions about incorporating natural products into their healthcare routines, while also fostering a sense of ownership over their well-being [23, 73].

**2. Collaboration is Key:** Fostering collaboration between traditional healers, scientists, and healthcare professionals is key. This will bridge the gap between traditional knowledge and evidence-based medicine, leading to a more comprehensive approach to managing metabolic diseases [73, 75].

**3. Collaboration and Sustainability: A Path Forward**

Sustainable harvesting practices are crucial to ensure the long-term availability of these natural resources. Collaboration between communities, scientists, and government agencies is key to achieving this; imagine Ugandan farmers cultivating medicinal plants using sustainable methods, ensuring a steady supply for research and patient care.

By addressing these considerations and fostering collaboration, Uganda can bridge the gap between traditional knowledge and modern medicine. This integrative approach holds the promise of empowering Ugandans to take charge of their health and create a healthier future for generations to come.

## LIST OF ABBREVIATIONS

AMPK: Adenosine Monophosphate-Activated Protein Kinase
DM: Diabetes Mellitus
IRS: Insulin Receptor Substrate
LMICs: Low and Middle-Income Countries
SLns: Sesquiterpene Lactones
